# Rho-dependent transcription termination in bacteria recycles RNA polymerases stalled at DNA lesions

**DOI:** 10.1038/s41467-019-09146-5

**Published:** 2019-03-14

**Authors:** Sriyans Jain, Richa Gupta, Ranjan Sen

**Affiliations:** Laboratory of Transcription, Center for DNA Fingerprinting and Diagnostics, Tuljaguda Complex, 4-1-714 Mouzamjahi Road, Nampally, Hyderabad 500 001 India

## Abstract

In bacteria, transcription-coupled repair of DNA lesions initiates after the Mfd protein removes RNA polymerases (RNAPs) stalled at the lesions. The bacterial RNA helicase, Rho, is a transcription termination protein that dislodges the elongation complexes. Here, we show that Rho dislodges the stalled RNAPs at DNA lesions. Strains defective in both Rho and Mfd are susceptible to DNA-damaging agents and are inefficient in repairing or propagating UV-damaged DNA. In vitro transcription assays show that Rho dissociates the stalled elongation complexes at the DNA lesions. We conclude that Rho-dependent termination recycles stalled RNAPs, which might facilitate DNA repair and other DNA-dependent processes essential for bacterial cell survival. We surmise that Rho might compete with, or augment, the Mfd function.

## Abstract (web summary)

In bacteria, the Rho protein dislodges elongation complexes to terminate transcription, and the Mfd protein removes RNA polymerases (RNAPs) stalled at DNA lesions. Here, Jain et al. show that Rho also dissociates stalled RNAPs at DNA lesions, which may facilitate DNA repair and other DNA-dependent processes.

## Introduction

In bacteria, the transcription process ends via two types of termination pathways, namely, intrinsic or factor-independent and Rho-dependent termination^1^. In *E. coli*, a large number of transcription termination events are Rho-dependent^1^. Rho is a homo-hexameric RNA-dependent ATPase that binds to nascent RNA by recognizing the *rut* site (rho utilization site; a C-rich unstructured stretch of the nascent RNA) and uses the energy obtained from the ATP hydrolysis to translocate along the RNA. Eventually it catches up the elongation complex (EC) and dissociates it^2,3^. Transcription elongation factor, NusG, interacts with Rho and stimulates the termination process^2,3^. Recent studies have revealed that by the virtue of its RNA-binding property and due to the all-pervading nature of the Rho-dependent termination, the Rho protein is found to be involved in the various physiological processes^3–5^.

During the bacterial growth, its DNA can be damaged by several extraneous physical (radiations) or chemical factors (hazardous chemicals or metabolites). To cope up with these stresses and to maintain the integrity of the DNA, bacteria have evolved the base excision repair (BER) system to remove single base aberrations and the nucleotide excision repair (NER) to remove large DNA lesions and the abasic sites^6–10^. NER initiates with the recognition of the DNA damage site by UvrAB complex following which the UvrA protein dissociates from the complex leaving behind the UvrB that recruits a nuclease, UvrC. UvrC cleaves the DNA fragment on the either side of the DNA lesions. UvrD, a helicase, unwinds the excised fragment and removes it. Later, the DNA polymerase fills in the gap and the DNA ligase seals the newly synthesized DNA to complete the repair process (see figure S1). In bacteria, NER is classified as global genomic repair (GGR) and transcription coupled repair (TCR) processes^11^. GGR pathway is a general DNA repair pathway that acts on the random genomic lesions, whereas the TCR is a DNA repair pathway that removes lesions from the transcribed strand of the expressed genes. In the latter process, the elongating RNAP stalls at the DNA lesions and acts like a signal for the recruitment of the NER-machinery to the damaged site. Mfd, a DNA translocase, dislodges stalled RNAP in order to expose the DNA lesions, and also has the ability to recruit the NER-protein, UvrA, through its N-terminal UvrB homology domain to initiate the repair process (supplementary figure 1)^12–14^. A whole body of literature has provided evidence that Mfd plays a pivotal role in initiation of the TCR, especially in repairing the damages of the transcribing strand^6,15–18^. However, in an alternative model, it has been claimed that a Mfd-independent pathway is also operational to dislodge the RNAP, where UvrD together with NusA induce the backtracking of the stalled ECs so that the DNA lesions are exposed to the other Uvr proteins^19,20^.

The transcription elongation is a discontinuous process. Depending on the DNA sequence and structures, the transcription elongation slows down or the RNAP stalls at the specific pause sites, which has various physiological consequences^21^. Besides this, elongating RNAPs stall at DNA damaged sites that are caused by physical and chemical agents^22–25^. Stalled EC, not only masks the damaged sites from the DNA repair but also could cause general hindrance to several DNA-dependent processes, which in turn would cause lethality. Therefore, it is essential to remove stalled RNAP from these DNA lesions.

**Fig. 1:**
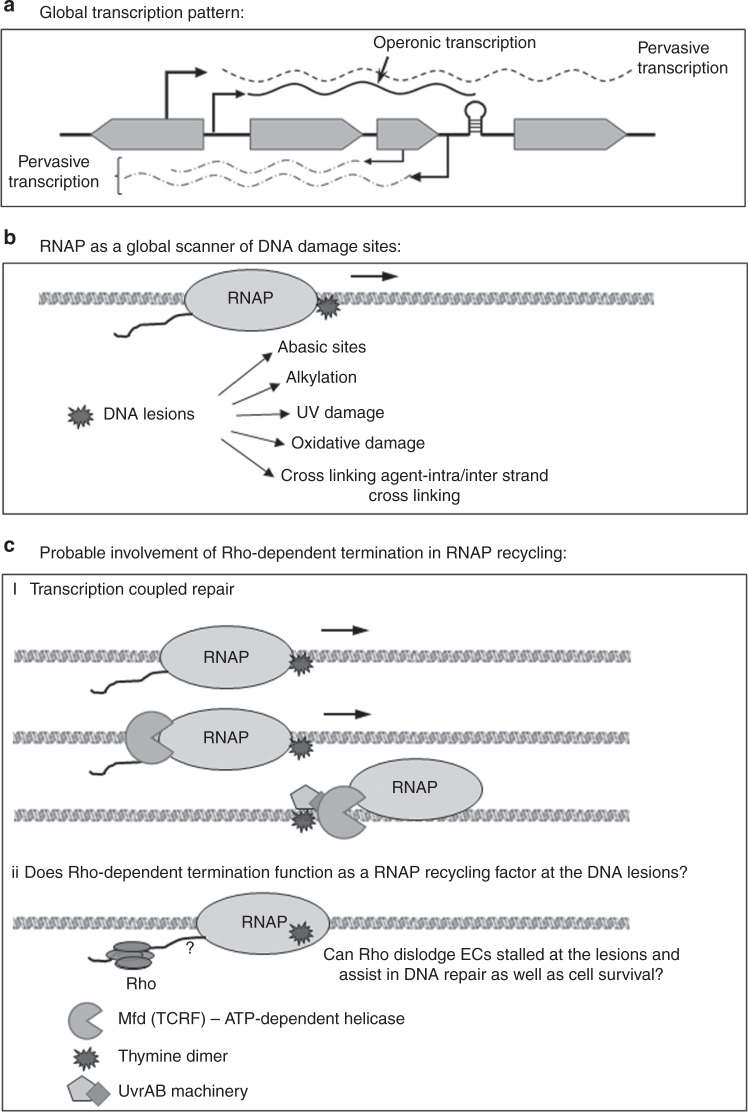
Hypothesis of connecting Rho-dependent termination and NER*:* Cartoons depicting (**a**) operonic (transcription starting from the promotor region of a gene) and pervasive (transcription starting from other cryptic start sites) transcription. **b** RNAP as a global scanner for DNA damage: By virtue of the pervasive as well as the operonic transcriptions, RNAP could encounter the DNA lesions in a genome-wide fashion, and thus could act as a global sensor for the DNA damages. **c** Probable mode of invoking NER processes by Rho by displacing the stalled transcription elongation complexes at the lesions. In this regard, it resembles the RNAP-dislodging function of Mfd

In a rapidly growing cell most of the RNA polymerases (RNAPs) are engaged in both the operonic and the non-operonic or the pervasive transcriptions (Fig. 1a)^26,27^. Hence, the elongating RNAP is thought to function as a global scanner of the DNA lesions (Fig. 1b)^12,20^. *E. coli* has ~1400 Rho molecules, which is nearly equimolar to the number of the elongating RNAPs ECs (~1250 per cell) in a cell^1^. So, it is likely that a significant fraction of the ECs will be the target of the Rho, especially those engaged in the non-operonic transcriptions. Consistent with that idea, recently it has been shown that one of the major roles of Rho is to prevent the pervasive transcription^28^. Earlier, we have shown that Rho can dislodge stalled ECs in vitro^23^. Hence, we hypothesized that Rho, by inducing transcription termination, is capable of recycling the RNAP stalled at the DNA lesions and thereby, might facilitate the repair of the damaged sites by unmasking them to the DNA repair machineries, and in this regard, it resembles the RNAP displacement activity of the Mfd (Fig. 1c). Failure of the Rho-mediated recycling of RNAPs would lead to lethality.

Here, we show that Rho mutants and NusG mutants (NusG is a transcription elongation factor that interacts with Rho and stimulates the termination process) exhibit synthetic growth defects with *mfd*. The *E. coli* strains carrying these mutations are more sensitive towards different DNA damaging agents and less efficient in propagating UV-damaged plasmid DNA. The Rho protein is efficient in releasing RNA from stalled ECs at the DNA lesions in in vitro transcription assays in a similar fashion as the Mfd protein. We conclude that Rho-dependent termination is capable of recycling RNAPs stalled at DNA lesions, thereby facilitating DNA repair and other DNA-dependent processes. Failure of this recycling leads to reduced survival of *E.coli*. We propose that Rho might either compete or augment the function of Mfd in vivo.

## Results

### Hypothesis

If Rho-dependent transcription termination process is involved in dislodging the ECs stalled at the DNA lesions and recycling the RNAPs, then in the presence of Rho mutants, defective for the termination function, should affect the DNA-dependent processes like DNA repair and the strains having these mutants should exhibit higher sensitivity towards the DNA-damaging agents. Also, failure of the RNAP-recycling is likely to lead to the reduction of survival of the cells. Hence, we explored to exhibit these in vivo phenotypes of the strains having the Rho mutants.

### Growth defects of the rho mutants when NER genes are deleted

**Fig. 2:**
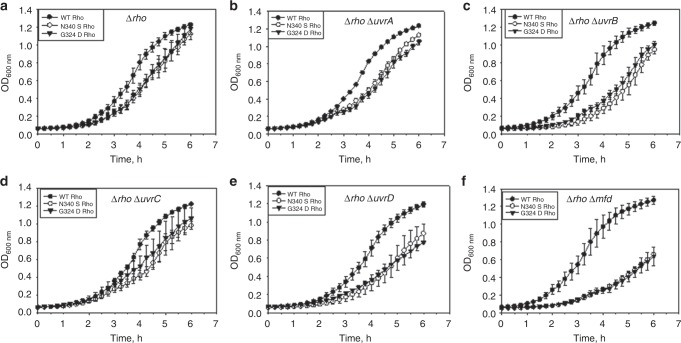
Synthetic growth defects of the Rho mutants with the different NER genes. **a**–**f** Growth curves showing the varied degrees of synthetic growth defects of the Rho mutant strains carrying deletions of individual NER genes. The WT or the mutant Rho proteins were supplied from the plasmids expressing WT or mutant *rho*. Error bars were calculated from the standard deviations obtained from at least three independent measurements. Source data are provided as a Source Data file

The synthetic growth defect in bacteria occurs when simultaneous perturbation of the two genes results in lethality or drastic reduction in the rate of growth. Determination of synthetic defects is a very useful tool to establish the genetic interactions between the two gene products. To assess the consequences of failure to dislodge the ECs stalled at the DNA lesions, we measured the synthetic growth defects of the Rho and NusG mutant strains in the absence of NER genes, products of the latter are involved in DNA lesions repair. In the WT *E. coli*, the NER genes are not essential during the log-phase growth (Fig. 2b–f; WT Rho plots)^29^. We employed synthetic growth defect assays by deleting each of the NER genes (*uvrA, uvrB, uvrC, uvrD* and *mfd;* see Methods section for the deletion procedures) individually from the *E. coli* MG1655 strain expressing either WT or the *rho* (N340S, G324D) or *nusG* (L158Q, G146D) mutants. These Rho mutants are defective in ATPase as well as termination activities^30^, whereas the NusG mutants are defective for the Rho-binding^31^. We observed the following. (I) When the *uvrA, uvrB, uvrC*, and *uvrD* (Fig. 2a–e) were deleted from the strains expressing the Rho mutants, milder growth defects were visible. (II) When *mfd* was deleted from the Rho mutants, the growth rate was reduced significantly (Fig. 2f). (III) When the strains were expressing the NusG mutants, the synthetic defects were less severe, in all the cases, except that for the *mfd* (data not shown). These synthetic effects were specific to the NER genes as the deletion of an unrelated gene, *uhpt*, did not elicit similar response (supplementary figure 2b).

These results indicated that Rho-dependent termination might be involved in dislodging the ECs stalled at the lesions and the process is genetically connected to the Mfd function. As Mfd function becomes most essential when Rho-dependent termination process is compromised, both of them might function in synergy.

### Sensitivity of the rho mutant strains to the DNA-damaging agents

As the dislodging of the EC and the unmasking of the DNA lesions is essential for the initiation of the repair process, it is likely that the *E. coli* MG1655 strains expressing the Rho mutants would be sensitive to the chemical (Mitomycin C and Cisplatin) or physical (UV-dose) DNA-damaging agents that also invokes NER response^32^. These agents create covalent adducts to the DNA that blocks the progression of the transcription EC^22,23^.

**Fig. 3:**
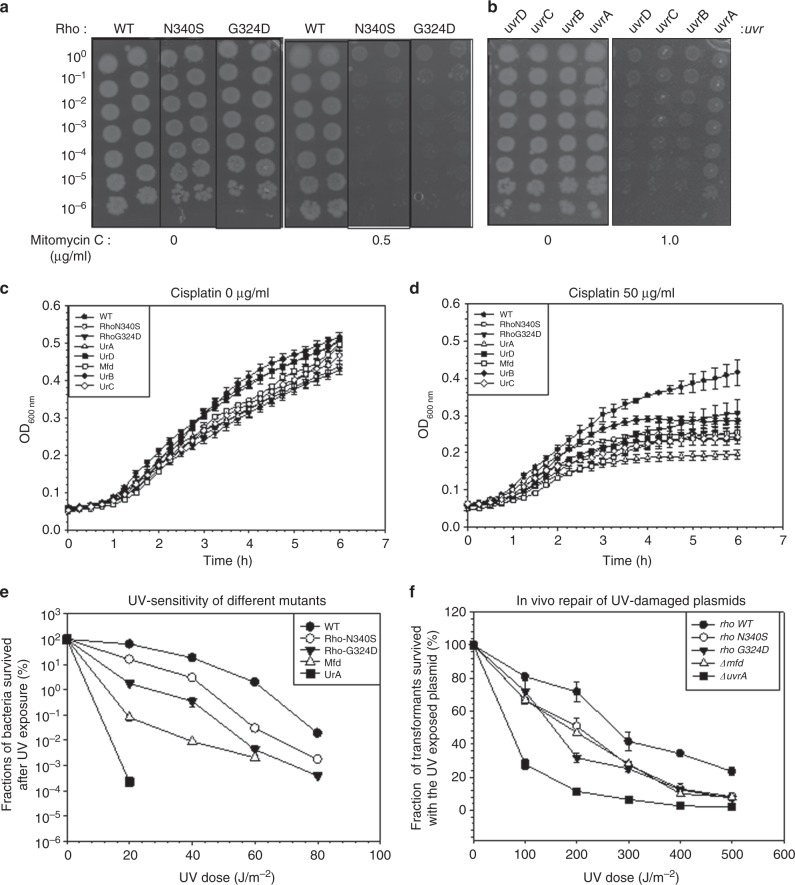
Sensitivity of *E. coli* MG1655 strains expressing different Rho mutants and having deletions in each of the NER genes towards the NER-inducing agents. **a** and **b** Serial dilution of mid-log phase culture of different strains were spotted on the LB plates containing indicated concentrations of Mitomycin C. Rho mutants were more sensitive towards Mitomycin C (0.5 µg/ml) as compared to the strains deleted for NER genes (1 µg/ml). **c**, **d** Growth curves showing the effect of Cisplatin on different strains as indicated. Rho mutants and various uvr deletion strains showed high sensitivity towards cisplatin compared to the WT strain. **e** Effects of different UV doses on the survival of the MG1655 strains having indicated mutations. **f** In vivo repair of UV-damaged plasmids in different strains. The plasmid pBR322 exposed to the increasing doses of UV was transformed to the strains either expressing mutant rho or are deleted for either uvrA or mfd. Fractions of number of transformants obtained were plotted against the UV-dose used to damage the plasmid in vitro. 4–5 replicates of the same experiment were performed to calculate the standard error of mean. Source data are provided as a Source Data file

Different derivatives of the MG1655 strains (see Fig. 3a, b) were grown in the presence of various concentrations of Mitomycin C. We observed that the strains expressing the Rho mutants, N340S and G324D, were highly sensitive to Mitomycin C even at a very low concentration (Fig. 3a). Similar sensitivity is observed for the strains devoid of the NER genes, *uvrA, uvrB, uvrC*, and *uvrD* (Fig. 3b). Next, we monitored the growth of different strains in the absence and presence of Cisplatin. Consistent with the Mitomycin-C effect, the said Rho mutants were also sensitive to this chemical like the *uvr*^*−*^ strains (Fig. 3c, d).

If the NER pathway is impaired, the *E. coli* becomes highly sensitive to the UV-radiation^14,33^. The initiation of the NER pathway depends on the dislodging of the ECs stalled at the UV-dose-induced thymine dimers. To further confirm the involvement of Rho-dependent termination in removing these stalled complexes, we monitored the UV-dose sensitivity of the strains expressing the Rho mutants, as well as the strains having deletions in the *uvrA, uvrD*, and *mfd*. The *uvrA*^*−*^ and *uvrD*^*−*^ strains showed extreme sensitivity, whereas the *mfd*^*−*^ strains were little less sensitive (Fig. 3e and Supplementary figure 3)^33^. The strains with *rho* mutants showed significant sensitivity towards the exposure to the UV-doses that is comparable to that observed for the *mfd*^*−*^ strain (Fig. 3e). In an old study, a Rho mutant, *rho15*, bearing strain was observed to be UV-sensitive, but the phenomenon was not linked to the NER process^34^.

Next, we measured the in vivo DNA repair proficiencies of the aforementioned strains expressing Rho mutants and compared them with those having *uvrA* or *mfd* deletions, which is a direct measurement of the involvement of Rho-dependent termination in invoking and facilitating the NER pathway (Fig. 3f)^35,36^. A plasmid DNA was damaged by exposing to the increasing doses of UV, and the in vivo DNA repair ability of the different strains were assessed by counting the number of transformants on the plates formed after the transformation with this damaged plasmid. The transformants will only form if the UV-damaged plasmid DNA is efficiently repaired in vivo. Figure 3f showed that DNA damage repair efficiency is very poor in the *uvrA*^*−*^ strain, whereas strains having the *mfd* deletion or expressing the Rho mutants are also defective in repairing the DNA lesions, albeit with a lesser severity, but the extent of this severity is significantly higher than the WT strain. However, we cannot rule out the possibility that the Rho mutants affected the DNA propagation step(s) instead of the DNA repair step(s), because the former would also have a similar manifestations of poor survival of the transformants.

Sensitivity of the strains expressing mutant Rho towards the physical and the chemical agents that induces NER as well as TCR, and their inability to efficiently repair the damages caused by UV-radiation to a plasmid DNA strongly suggest that the dislodging of the RNAP stalled at the DNA lesions by the Rho-dependent termination facilitates the initiation of the NER pathway. Failure to recycle the stalled RNAP by the Rho mutants led to reduced survival of the cells. The aforementioned several lines of evidences also suggest that the severity of phenotypes induced by the Rho-mutants is comparable to those observed when the strains are devoid of the Mfd protein.

### In vivo combinatorial effect of the Rho and the Mfd mutants

**Fig. 4:**
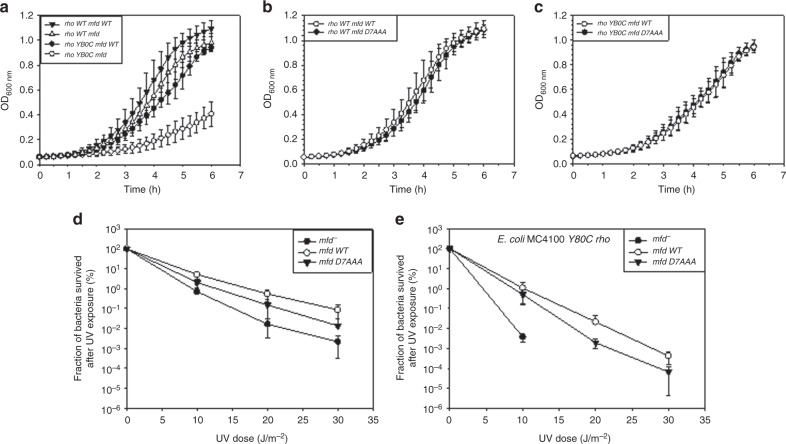
Combinatorial effects of the Rho mutants and either the Mfd deletion or the Mfd mutant. **a** Growth comparison of *E. coli* MC4100 carrying WT or mutant (Y80C) Rho in the presence or absence of the *mfd*. In combination with rho Y80C, the deletion of *mfd*, induces severe slowing down of the bacterial growth. **b** and **c** are showing the growth curves of MC4100 WT *rho* and MC4100 Y80C*rho* strains expressing Mfd D7AAA. The mutant Mfd suppressed the growth defects of Δ*mfd* strain. **d** and **e** UV sensitivity of *E. coli* MC4100 WT *rho* and MC4100 Y80C *rho* strains having the *mfd* mutations or deletions as indicated. Δ*mfd* strains were highly sensitive to the UV doses whereas the strain expressing the Mfd D7AAA showed a moderate sensitivity as compared to the WT Mfd. Error bars were calculated from the standard deviation obtained from at least three independent measurements. Source data are provided as a Source Data file

To further provide evidences that the Rho-dependent termination and the Mfd functions belong to the same pathway(s) of the NER process, we wanted to observe the combinatorial effect on the NER when both these functions are compromised simultaneously. In Fig. 2, we have observed that deletion of *mfd* in the presence of two very defective Rho mutants, N340S and G324D, had a severe growth defect and the resultant strains are too sick to be amenable for further in vivo assays. Hence, we used an *E. coli* strain (RS1714) that expresses a milder Rho mutant, Y80C^37^. We transformed the strains having a WT (RS257) or Y80C *rho* (RS1714) in the chromosome either with the vector pBR322 alone or expressing the WT *mfd*. The growth of these resultant strains were monitored after deleting the chromosomal *mfd* (Fig. 4a). Healthy looking colonies and the good growth rates were observed in all the cases except for the Y80C Rho and *Δmfd* combination.

Aforementioned strains expressing different variants of the Rho in the presence and absence of the Mfd protein were subjected to various UV doses (Fig. 4d, e) and their survival were assessed. Severe UV sensitivity was observed for the strain that expresses Y80C Rho mutant in the absence of the Mfd protein (Fig. 4e). The UV-sensitivities were milder when either Rho or Mfd functions were separately disabled or were functioning less efficiently (see also Fig. 3e). This additive effect, when both the Rho and Mfd functions are compromised, on the UV-sensitivity, as well as on the cell growth further suggest that the presence of one of them in the cell is essential for the invocation of the NER as well as the survival of the cells.

In the NER, Mfd performs two functions: (i) recruit UvrA to the damaged site and (ii) displace the stalled RNAP from the damaged site. Rho is capable of only displacing the stalled EC^38^. So, if the stalled ECs are displaced by Rho, instead of Mfd, how could the Uvr proteins be recruited to the DNA damaged site? If the Mfd and the Rho function in synergy, a constitutively acting Mfd mutant (capable of functioning without interacting with RNAP) is likely to suppress the synthetic growth defects caused by the Rho mutants and the *mfd* deletion. A Mfd D7-domain mutant, D7AAA (E1045A, D1048A, R1049A), is a de-repressed Mfd protein that is constitutively active even without interacting with the RNAP and has mild effect on TCR^39^. As was expected, we observed that this mutant did not exhibit synthetic growth defect with the Y80C Rho mutant (Fig. 4b, c) and the strains expressing both D7AAA Mfd and Y80C Rho suppressed the severe UV-sensitivity that was observed when *mfd* was deleted (Fig. 4e). This result may suggest that Rho and Mfd function cooperatively to dislodge the ECs stalled at the DNA lesion sites.

### Rho releases RNA from the stalled ECs at the DNA lesions

By the virtue of the EC-dislodging properties of Rho, similar phenotypes of the Rho mutants like the *mfd*^*−*^ strains and high mutual dependence of both the *rho* and *mfd* during the in vivo DNA repair process, strongly indicate that Rho protein is capable of removing the stalled ECs from the DNA lesions in a similar fashion as the Mfd protein.

**Fig. 5:**
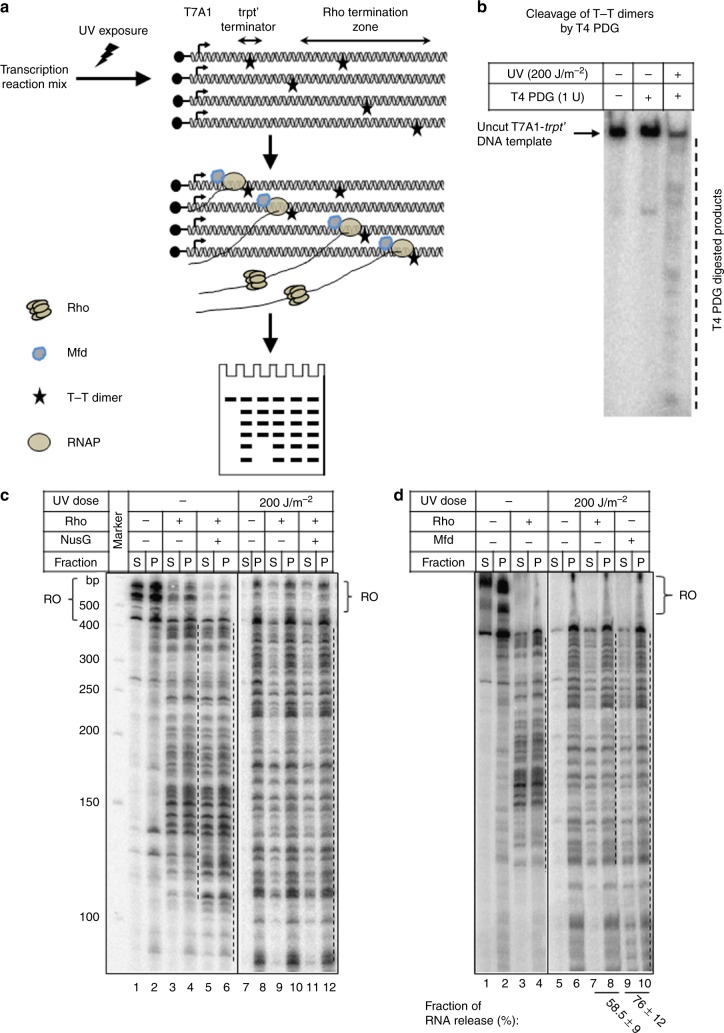
In vitro transcription assays to measure RNA release. **a** Cartoon showing the DNA template used for the study. Promoter and the terminator regions are indicated. T–T dimers, generated from the UV-exposure, formed at various sites on the template strands are indicated by (*). ECs with variable RNA chain length are shown to get stalled at the lesions that are located at the proximal most sites from the transcription stat-site. Mfd or Rho dislodges these stalled ECs and release RNA molecules of variable chain length that are analyzed by gel-electrophoresis. Rho loads onto these RNA once the latter reaches the critical lengths of 60–90 nt. Rho and Mfd proteins are indicated. **b** Autoradiogram showing selective degradation of UV-irradiated DNA by T4-PDG. Radioactive labeled transcription template DNA, with or without UV exposure, was subjected to the cleavage with T4-PDG. **c** and **d** Autoradiograms showing in vitro transcription performed on the immobilized DNA templates under different conditions as indicated. S denotes half of the RNA release in the supernatant and P denotes the other half of RNA and the total pellet. Transcripts those reached at the end of the template are denoted as run-off (RO). Zones of RNA release are shown next to the autoradiograms by dashed lines. Fractions of RNA release is calculated as: 2S/([S]+[S+P]). Amounts of RNAP, DNA, Rho, and Mfd were 25, 10, 100, and 100 nM, respectively

To directly test this proposition, we designed Mfd-induced and Rho-induced in vitro RNA release (a measure of RNAP recycling) assays from the ECs stalled at the DNA lesions (Fig. 5a). We used a linearized 5′-biotinylated DNA having a strong T7A1 promoter. On this template, transcription initiates from this promoter and elongates through a Rho-dependent terminator region, *trpt’*, and the template is immobilized on the streptavidin-coated magnetic beads via a biotin linkage. This DNA template was exposed to UV-radiation (200 J m^−2^) that resulted in the formation of several T–T dimer (thymine-dimer adducts; stars in Fig. 5a). We confirmed the existence of these adducts by the sensitivity of this damaged template to an enzyme called, T4-PDG (from bacteriophage T4, pyrimidine dimer glycosylase; NEB) (Fig. 5b, third lane). We then proceeded with the transcription reactions on this template, both in the presence and absence of either Rho or Mfd proteins (Fig. 5c, d).

Figure 5c depicts the comparison of the Rho-induced RNA release (immobilized templates were used so that the released RNA could be measured in the supernatant, S) in the termination zone of an un-irradiated DNA (left panel) and the T–T containing template (right panel), whereas the comparison of the Rho-induced and Mfd-induced RNA release are shown in Fig. 5d, see also supplementary figure 6a). We observed the following. (1) Several stalled ECs are formed when the DNA is irradiated (Fig. 5c, lane 8 and 5D lane 6). (2) Rho is capable of releasing RNA from the stalled ECs those are in its termination zone only (compare both the panels of Fig. 5c and also supplementary figure 6a), whereas Mfd releases RNA from all the stalled ECs (Fig. 5d, lanes 9 and 10). (3) Presence of NusG did not change the RNA release pattern of the Rho protein. We also compared the RNA release efficiency of these two proteins from the stalled ECs formed either due to the presence of low concentrations of NTPs (supplementary figure 4a) or because of a protein roadblock (supplementary figure 4b), and found that both these proteins have comparable functions. We concluded that like Mfd, Rho is also capable of dislodging stalled ECs at the DNA lesions. However, Rho is not capable of recruiting Uvr proteins to the damaged sites.

### Comparison of RNA release efficiencies between Mfd and Rho

We have earlier reported that the Rho prefers transcriptionally active stalled ECs to release RNA and the RNA-release kinetics is significantly slower from the arrested or backtracked ECs^38^. The covalent adducts of T–T dimers are quite stable and induces backtracking as was evident from the sensitivity of the stalled ECs at the lesions towards the GreB (Supplementary figures 5b and 5c). In the previous section, we observed in the steady-state measurements that the Rho-induced RNA release efficiency from these stalled ECs was ~20% less than what was observed in the presence of the Mfd. Hence, we looked into the RNA release kinetics from the ECs stalled at the T–T dimers under various in vitro conditions to assess the mechanistic differences between the mode of functions of these two terminators.

**Fig. 6:**
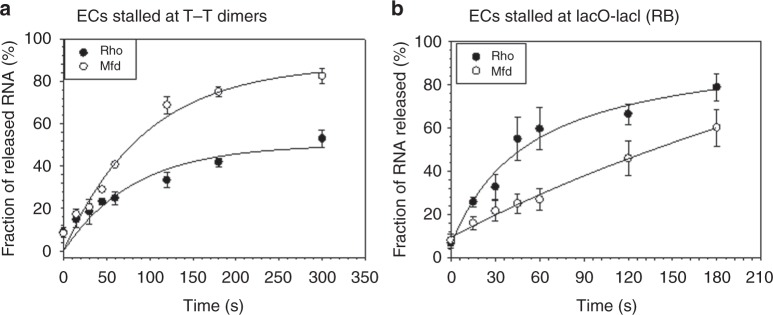
Kinetics of Rho and Mfd induced RNA release from the stalled ECs at the T–T dimers*:* Fractions of either Rho-induced or Mfd-induced RNA released from the ECs stalled at the T–T dimers (**a**) or at a LacI-LacO road-block site (**b**) were plotted against the time. Curve fittings were performed by using exponential increase equation of the form, *y* = *a* (1−*e*^−^^*bx*^), where “*a*” is amplitude and “*b*” is the rate. The fraction of RNA release in (**a**) were calculated from the band intensities indicated by * in supplementary figure 6a, and those in (**b**) are from the RB bands in supplementary figure 6b. Error bars were calculated from the standard deviations obtained from at least three independent measurements. Source data are provided as a Source Data file

We formed two types of stalled ECs; one at the T–T dimer lesions (similar to those in Fig. 5 and in supplementary figure 6a) and the another one at LacI bound to a lac operator site (see supplementary figure 6b). The latter one remains transcriptionally active and is a good substrate for Rho to act upon as was shown by us earlier^38^. We calculated the average of the fractions of RNA release from the ECs stalled at five different T–T dimers inside the termination zone both in the presence and the absence of either Rho or Mfd (denoted as * in supplementary figure 6a) proteins. In separate assays, we measured the fractions of RNA release from the stalled EC (RB) at a LacI bound site on the template (supplementary figure 6b). We observed that the speed of RNA release is significantly faster from the stalled ECs at the T–T dimer in the presence of Mfd (Fig. 6a), whereas Rho induces very fast RNA release from the transcriptionally active RB (Fig. 6b)^38^. Therefore, already backtracked ECs at the T–T DNA lesions are not very good substrates for the Rho as compared to the Mfd. So, to efficiently remove RNAPs from these lesions in vivo, Rho should act instantaneously as soon as the transcription elongation is blocked or should be dependent on the actions of the transcription anti-arrest factors like, GreA and GreB. However, Mfd is capable of performing the same function independently of these anti-arrest factors.

## Discussion

The faster removal of the stalled ECs at the DNA lesions and presentations of the damaged sites to the NER enzymes is the most important rate-limiting step(s) for the cascade of TCR (a special form of NER) reactions to follow it (supplementary figure 1a)^8,14^. Slow removal of the ECs could also lead to replication–transcription collision affecting the overall genome integrity^40,41^. Here, using tools to study synthetic growth defects, in vivo assays for the sensitivity to the DNA damaging agents and in vitro RNA and RNAP release assays, we have proved that Rho-dependent termination is capable of recycling RNAPs from the ECs stalled at the damaged sites. This recycling of RNAPs by Rho in turn invokes NER, prevents replication–transcription collision and other DNA-dependent processes, which is essential for the survival of the bacteria. We have furnished the following lines of evidences to establish our claim. (1) Rho and NusG mutants compromised for the transcription termination functions cause mild (for the *uvrA, uvrB, uvrC*, and *uvrD*) to severe (for *mfd*) growth defects in combination with the deletion of different NER genes (Fig.2). (2) Strains expressing Rho mutants are significantly sensitive to DNA-damage-inducing physical and chemical agents (Fig. 3a–e), and are inefficient in repairing the plasmid DNA damages and/or its propagation in vivo (Fig. 3f). (3) Similar to Mfd, Rho is capable of releasing the nascent RNA as well as dislodging the stalled ECs at the T–T dimers (Fig. 5). (4) Combination of a Rho mutant and the *mfd* deletion amplified the sensitivity of the strains towards UV by several fold as compared to that observed when only Mfd function was compromised (Fig. 4). Aforementioned results also allow us to propose that Rho either augments or competes with Mfd when the latter is present, and it may complement the function when the latter is absent. Since a constitutively functioning derivative of Mfd was able to suppress the combinatorial defects of Rho mutants and the Mfd deletion (Fig. 4), there is a possibility that these two terminators might function in tandem to recycle the trapped RNAPs and unmask the DNA lesions for the subsequent repair steps to occur. Because the removal of the ECs and the exposure of the DNA lesions to the Uvr proteins have to be performed with extreme efficiency and speed, synergy between them would be hugely beneficial to the cells.

Even though the dislodging of the ECs from the DNA lesion sites are essential, surprisingly, Mfd, the only protein so far known to be responsible for this is not an essential protein in *E. coli*, and *mfd*^*−*^ strain has a milder sensitivity towards DNA-damaging agents (Fig. 3)^14,33^. To solve this puzzle, recently it was proposed that UvrD, a DNA helicase and a component of the TCR pathway, induces backward movement of the RNAP and opens up the access of the DNA lesions to the other Uvr proteins^19,20^, thereby renders requirement of Mfd redundant. However, this claim was contested in the subsequent publications^15,16,42^. Here, we believe that inclusion of another terminator, the Rho protein, in recycling step(s) of the RNAPs from the DNA lesions, solves the puzzle of the less dependence of the *E. coli* on the Mfd protein. We propose that in the absence of the Mfd, Rho dislodges the stalled ECs and let the repair process continue unhindered. This is the major reasons for the observed synthetic growth defect when both the *rho* and *mfd* functions are compromised (Figs. 2a and  4). By the virtue of its termination properties, Rho facilitates the DNA repair process, thereby contributes immensely in the survival of the bacteria.

Although both the Rho and the Mfd proteins dislodge ECs, there are distinct mechanistic differences in the way they function. One of the major roles of Mfd in TCR is to recruit the UvrA protein to the damaged site (see supplementary figure 1), which is the most important step to assemble other Uvr proteins and initiate the subsequent reactions. Rho is not capable of recruiting the Uvr proteins, and hence, in the absence of Mfd, Rho technically cannot initiate the TCR process, instead it might invoke the GGR (see supplementary figure 1) path, where Uvr proteins are drawn to the exposed DNA lesions passively. However, in the presence of Mfd, both these terminators might function synergistically as described before.

In addition to the aforementioned limitation, Rho can only dislodge the ECs stalled at the DNA lesions that are inside the termination zone of a Rho terminator. There is an a priori requirement of a terminator region (including the Rho-loading sites) for the Rho protein to function, whereas the Mfd is more versatile and can act on any stalled ECs (see Fig. 5). The Rho is more efficient to dislodge the transcriptionally active ECs, whereas it appears that the Mfd has evolved to function preferentially on arrested and backtracked complexes (Fig. 6). This difference arises because one is a RNA helicase and the other is a DNA translocase. Rho dislodges the EC by pulling the RNA out of the active site either by the tethered tracking or by the simple tracking modes of its translocase activity along the RNA^2,3^ and the mechanism may also involve a direct collision of RNAP and Rho^38^. A transcriptionally active EC is likely to have a relatively unstable RNA:DNA hybrid because of its inherent dynamic nature, from which pulling out the mRNA might be easier than an arrested EC, where the RNA protrudes into the secondary channel (NTP entry channel)^1,43^ and have additional interactions inside this channel. The Mfd protein destabilizes the RNAP by forward translocating it along the DNA template^8^, thereby it is likely to be more effective on the arrested complexes at the DNA lesions. The Rho-dependent termination is a genome-wide phenomenon and majority of the operons are the targets of Rho^28,44,45^, therefore most of the ECs would be associated with the Rho rendering its availability to the DNA lesion sites.

In the synthetic growth defect assays, the *uvrD* deletion exhibited milder effect with the Rho and the NusG mutants (Fig. 2), indicating that both the Rho and the uvrD helicase may not be genetically connected during the repair process. In the mechanistic term, the DNA unwinding step performed by UvrD following the RNAP/RNA release is not dependent on the Rho function. Even if the UvrD is involved in pulling back RNAP from the DNA lesions site^19^, the Rho would function independently of this phenomenon. However, because the backtracked ECs are poor substrates for the Rho protein, it is likely to function more synergistically with the Mfd rather than with the UvrD.

Apparent less-specificity of the RNA sites (the *rut* sites) recognized by the Rho helicase subjects a wide range of operons as well as non-operonic regions to be its targets, thereby bringing the expression of a huge number of genes under the control of Rho. All the genomic data^37,44,46^ indicate that the inhibition of Rho induces expression of a whole set of new genes, which changes the physiology of the cell. Therefore, it is not surprising that Rho is involved in so many physiological processes^3,5^, and the present study adds one more crucial function of Rho to this list. The functions of this transcription terminator are an interesting example, where pleotropicity is a boon to the system and favored over the usual specificity that makes the hallmark of the biological systems. We surmise that the Rho protein functions as a “master-regulator” of various physiological processes of bacteria and should be considered as a potent drug target.

## Methods

### Materials

The reagents for the in vitro transcription reactions (Tris–Cl, MgCl_2_, KCl, DEPC-treated water) were purchased from Ambion. All the nucleotides were from GE healthcare. [α-^32^P] CTP was procured from Jonaki, BRIT, Hyderabad. The streptavidin-coated magnetic beads were from Promega. The restriction enzymes, the DNA ligase and the RNA polymerase were purchased from NEB, USA. The Taq DNA polymerase for synthesis of transcription template was from Roche Applied Sciences. All the plasmids used in this study are listed in Table 1. All the primers used in this study were synthesized by Eurofins Genomics India Pvt. Ltd. and are listed in Table 2. His-tagged versions of the Rho, NusG, and Mfd were purified using Ni^2+^-NTA agarose columns (Qiagen). Heparin-sepharose columns from GE were used as an additional step in the purification of the Rho protein.

**Table 1:** Plasmids used in the study

Plasmid names	Description	Reference
pHYD1201	*rho* subcloned from pHYD567 into *Hin*dIII-*Sal*I sites of pAM34 (pMB9; IPTG dependent replicon, Amp^R^)	^49^
pHYD751	*nusG*^+^ subcloned from pHYD547 into *Eco*RI-*Sal*I sites of pAM34 (pMB9; IPTG dependent replicon, Amp^R^)	^49^
pRS96	WT *rho* cloned at NdeI/XhoI site of pET21b, Amp^R^	^30^
pRS106	pT7A1 cloned at EcoRI/HindIII sites upstream of trpt cloned at HindIII/BamHI sites of pK8641, Amp^R^	^48^
pRS119	WT *nusG* cloned at NdeI/XhoI site of pET21b, Amp^R^	^30^
pRS614	*E. coli greB* cloned in pET21b at XhoI/NdeI sites, Amp^R^	Irina Artsimovitch
pRS316	Wt NusG cloned in pCL1920, Spec^R^,Strep^R^	^30^
pRS1049	NusG G146D in pCL1920 made by SDM of RS316, Spec^R^	^45^
pRS1856	NusG L158Q in pCL1920 made by SDM of RS316, Spec^R^	^45^
pRS317	*E. coli rho* cloned in pCL1920, Spec^R^	^30^
pRS725	Rho N340S in pCL1920, Spec^R^	^30^
pRS1106	Rho G324D in pCL1920, Spec^R^	^30^
pRS1807	*E. coli mfd* cloned at NheI/HindIII site of pET21a, Amp^R^	This study
pRS1931	*E. coli* WT *mfd* cloned along with its own promotor at the *EcoRI/HindIII* site of pBR322, Amp^R^	This study
pRS1933	pRS1931, Mfd D7AAA, Amp^R^	This study

**Table 2:** Oligonucleotides used in this study

Oligo name	Description
RS83	ATAAACTGCCAGGAATTGGGGATC; located upstream of T7A1 promoter of pRS106; Biotinylated at 5’ end
RSRK1	GTTTTCCCAGTCACGAC
RS177	GAATTGTGAGCGCTCACAATTCGGATATATATTAACAATTACCTG, reverse primer with lacO sequence downstream rut site of pRS106
RS1464	ATGGCTAGCATGCCTGAACAATATCGTTATACGC; forward primer with NheI site for cloning of *E. coli mfd* in pET21a
RS1465	TATAAGCTTAGCGATCGCGTTCTCTTCC; reverse primer with HindIII site for cloning of *E. coli mfd* in pET21a
RS1743	CAGATCGGTCATCAATGCGT; forward primer binding in *rho* upstream region
RS1744	AACAGAAACAGTGTCGTGAA; Reverse primer binding in *rho* downstream region
K1	CAGTCATAGCCGAATAGCCT; Kanamycin gene downstream reverse primer
RS1095	ATGTTGTGACCTCGGTTCC; binds to *uvrA* upstream region
RS1097	GATTGACAGCGGAGTTTACGC; binds to *uvrB* upstream region
RS1099	TGATGATCACCAAGGGCCAG; binds to *uvrC* upstream region
RS1101	ATTTCCCGGTTGGCATCTCT; binds to *uvrD* upstream region
RS1740	CATGAATTCAGCCGGGGCGATTCAATTTGC; binds to *mfd* upstream region, for cloning *mfd* gene with its own promoter
RS1741	TTTAAGCTTAAGCGATCGCGTTCTCTTCC; binds to *mfd* downstream region, for cloning *mfd* gene with its own promoter

### Bacterial strains

Bacterial strains used in this study are listed in Table 3. *E. coli* MG1655 was used as the parent strain for all the subsequent modifications. Since *rho* is an essential gene^29^, an exogenous copy of *rho* was provided through an IPTG-dependent shelter plasmid pHYD1201 in the strain RS1305 (*E. coli* MG1655 *Δrho*). RS1309 (*E. coli* MG1655 *Δrho Δrac*) was generated by transducing RS1305 with P1 lysate carrying the deletion of *rac*. When required, the NER genes (*mfd, uvrA, uvrB, uvrC*, and *uvrD*) and an unrelated gene, *uhpT*, were deleted from the RS1309 carrying the shelter plasmid, pHYD1201 by the P1 transduction method^47^.

**Table 3:** Bacterial strains

Strain no.	Description	Reference
RS1099	*E. coli* MG1655 *ΔnusG Δrac*, Carrying shelter plasmid pHYD751 (Amp^R^)	^45, 50^
RS1305	*E. coli* MG1655 *Δrho*, Carrying shelter plasmid pHYD1201 (Amp^R^)	^50^
RS1309	*E. coli* MG1655 *Δrho Δrac*, Carrying shelter plasmid pHYD1201 (Amp^R^)	^50^
RS1905	*E. coli* MG1655 *Δrho Δrac ΔuvrA*, Carrying shelter plasmid pHYD1201 (Amp^R^)	This study
RS1906	*E. coli* MG1655 *Δrho Δrac ΔuvrB*, Carrying shelter plasmid pHYD1201 (Amp^R^)	This study
RS1907	*E. coli* MG1655 *Δrho Δrac ΔuvrC*, Carrying shelter plasmid pHYD1201 (Amp^R^)	This study
RS1908	*E. coli* MG1655 *Δrho Δrac Δmfd*, Carrying shelter plasmid pHYD1201 (Amp^R^)	This study
RS1811	*E. coli* MG1655 *Δrho Δrac ΔuvrD*, Carrying shelter plasmid pHYD1201 (Amp^R^)	This study
RS257	*E. coli* MC4100 *galEP3*	^49^
RS1714	*E. coli* MC4100 *galEP3 rhoY80C*	
RS1927	*E. coli* MC4100 *galEP3 Δmfd* (Kan^R^)	This study
RS1928	*E. coli* MC4100 *galEP3 rhoY80C Δmfd* (Kan^R^)	This study

In order to check different combinatorial effects (Fig. 4) of deletion or point mutants of *mfd* in the presence of compromised Rho-functions, we have used *E.coli* MC4100 having WT (RS257) or *Y80C* (RS1714) *rho* in the chromosome. This strain was made *Δmfd* by P1 transduction as above. When required, WT or mutant Mfd proteins were expressed from a pBR322 plasmid.

Similarly, RS1099 (*E. coli* MG1655 *ΔnusG Δrac*; NusG supplied from a shelter plasmid pHYD751) was constructed by deleting chromosomal copy of *nusG* by P1 transduction. RS1099 was subsequently transformed with pCL1920 carrying either WT or mutant *nusG* (L158Q, G146D) and cured of the shelter plasmid, pHYD751.

### Assessment of synthetic growth defects

To examine the synthetic growth defects of the different NER genes (*mfd, uvrA, uvrB, uvrC*, and *uvrD*) with the *rho* mutants, these genes were deleted individually by P1 transduction method in the strain RS1309 carrying a shelter plasmid (pHYD1201) expressing the WT *rho*. The specificity of the transduction is shown by the gene-specific PCR method in the Supplementary figure 2a. Since the *rho* and *uvrD* genes are closely linked, transductants with uvrD lysates that did not co-transduce WT *rho* were screened by PCR (supplementary figure 2c). Strains that carry deletion of both the *rho* and each of the NER genes, were transformed with plasmid pCL1920 expressing either WT or mutant *rho* and then the strains were cured of the shelter plasmid, pHYD1201. To assess the growth defects of strains carrying deletion of NER genes in combination with *rho* mutation, overnight cultures of these strains were sub-cultured and the growth curves were obtained by growing in microtitre plates (Fig. 2a–f).

To examine the synthetic growth defects of the different NER genes (*mfd, uvrA, uvrB, uvrC* and *uvrD*) in the presence of the *nusG* mutants, these genes were deleted one at a time from the derivatives of strain RS1099 by P1 transduction. The number of transductants were directly measured from the transduction plates to assess the synthetic defects. The synthetic growth defects of an unrelated gene, *uhpT*, in combination with *rho* mutants was also examined in the same way.

To check the synthetic growth defects of MC4100 strain having a combination of Rho mutant and the *mfd* deletion or point mutants, the MC4100*Δmfd* strain expressing WT (RS1927) or Y80C (RS1928) Rho proteins were transformed with pBR322 plasmid (for *Δmfd*) or its derivatives expressing the WT or D7AAA *mfd*^39^. Growth curves of the resultant strains were obtained by growing them in the microtitre plates and by real-time monitoring of the OD_600_.

### Growth assays in the presence of the DNA damaging agents

To examine the efficiency of DNA repair machinery in the presence of defective Rho protein and the various NER mutants, the derivatives of RS1309 were exposed to various DNA damaging agents and effect on their growth was measured. (i) Mitomycin C: Overnight cultures of different derivatives of the RS1309 strains were serially diluted in the fresh LB broths. 5 µl cultures were spotted on LB agar plates containing different concentrations (0, 0.5, and 1 µg/ml) of mitomycin-C and incubated overnight at 37 °C. (ii) UV sensitivity: Serial dilutions of overnight grown cultures of the derivatives of the RS1309 were spreaded evenly on LB agar plates and incubated for 30 min at 25 °C. These plates were then exposed to various doses (0, 20, 40, 60, and 80 J m^−2^) of 254 nm UV light in an UV crosslinker (CL-1000 UV Crosslinker, Ultra-Violet Products Ltd., Cambridge, UK). Following the UV exposure, plates were covered with aluminum foil and incubated overnight at 37 °C. The fraction of surviving colonies relative to the colonies grown on the unexposed plates were calculated and were plotted against the UV-dose. UV-sensitivity assays of the MC4100 derivatives (Fig. 4) were also performed in the same way as above. (iii) Cisplatin sensitivity: 2 µl overnight cultures of the derivatives of the RS1309 were inoculated in 198 µl of LB broth containing different concentrations (0 and 50 µg/ml) of cisplatin in a microtitre plate. The growth was measured from the changes in the optical density at 600 nm at 37 °C in a Spectramax Microtitre plate reader (Molecular devices, USA). Optical densities were plotted against the time to generate the growth curves.

### Assessment of in vivo DNA repair

To determine the efficiency of in vivo DNA repair in the strains carrying different *rho* mutants and in the strains with *mfd* or *uvrA* deletion, we transformed them with 10 ng of plasmid pBR322 exposed to increasing doses (100–500 J m^*−*2^) of the UV light in vitro. The efficiency of in vivo DNA repair was determined by the fractions of transformants obtained for each strain with respect to the transformants of the same strain obtained with the unexposed plasmid and was plotted against the UV dose. As the variation in the number of transformants against each doses was high, we repeated the experiments at least five times to reduce the standard deviations and to get more statistically significant results.

### Transcription termination assays

(i) *RNA release assays*: To measure the efficacy of the Rho-dependent and Mfd-dependent RNA release from the ECs stalled at the thymine dimers (T–T) made along the template, a linear T7A1-trpt′ DNA template was synthesized by PCR amplification from the plasmid pRS106 using primers RS83 as the forward and RSRK-1 as the reverse primers^48^. On this template, the transcription initiates from a strong T7A1 promoter, and contains a T-less sequence in its first 22 bases so that in the absence of UTP in the reaction mixture, a 23-mer stalled elongation complex (EC_23_) could be formed. The DNA template was exposed to various UV doses in an UV crosslinker (CL-1000 UV Crosslinkers, Ultra-Violet Products Ltd., Cambridge, UK). We chose the UV dose of 200 J m^*−*2^ as it gave an optimum number of T–T dimers along the template. Formation of T–T dimer were determined from the sensitivity of the UV-exposed template to the enzyme T4-PDG (NEB, USA). The UV exposed templates were immobilized on the streptavidin-coated magnetic beads through the 5′-biotin and then used for the in vitro transcription assays. The EC_23_ was formed by mixing 5 nM DNA template, 25 nM RNA polymerase in the transcription buffer (25 mM Tris–HCl pH 8.0, 5 mM MgCl_2_, 50 mM KCl, 1 mM DTT, and 0.1 mg/ml of BSA) and 175 µM ApU, 5 µM GTP, 5 µM ATP, 2.5 µM CTP, and [α-^32^P]CTP (3000 Ci/mmol; Jonaki, BRIT, Hyderabad). The EC_23_ was then chased with 250 µM each of ATP, GTP, CTP, and UTP for 2 min. Under this experimental condition, a series of stalled ECs were observed to form on the UV-exposed templates. To these stalled complexes, either 100 nM of WT Rho or 100 nM Mfd were added and were incubated for 5 min. Released RNA was separated from the RNA bound to the EC by holding the tube against a magnetic stand. Half of the supernatant (S) was collected and other half of reaction mixture along with pellet (S+P) was subjected to phenol extraction. Both the samples were then mixed with equal volume of denaturing RNA loading dye, heated at 95 °C for 2 min and run on 8% Urea–PAGE. Amount of RNA released in each fraction was estimated by the signal intensity of the different bands in the supernatant and pellet fractions as measured by ImageQuant software (Molecular Dynamics, Inc.). Fraction of released RNA was then calculated as [2S]/([S]+[S+P]).

We also determined the efficiency of RNA released by the Rho or the Mfd from the ECs stalled due to the depletion of nucleotides or by a protein road-block. To pause transcription by nucleotide depletion, the EC_23_ was chased with only 2.5 µM NTPs. To study the RNA release from the EC stalled at a protein road block, we amplified the transcription template from plasmid pRS106 using primer RS83 as forward primer and RS177 as reverse primer. Primer RS177 carries LacO sequence at 3′-end to provide the binding site for LacI protein. To create the protein road-block, 300 nM LacI was added in the transcription reaction before the formation of the EC_23_.

### RNA release kinetic assays

RNA release kinetic assays were performed in a similar manner as described above except that the aliquots of reaction mixtures were taken out at different time points. The supernatant and the pellet fractions from the different aliquots were fractionated by holding the microfuge tubes against a magnetic stand and loaded onto a Urea–PAGE. The fractions of RNA released were calculated as above and plotted against time. To assess the extent of backtracking of the stalled ECs at the different T–T dimer sites, we added 500 nM GreB at different time points after formation of the ECs and removing the NTPs (supplementary figure 6a and b).

### Reporting summary

Further information on experimental design is available in the Nature Research Reporting Summary linked to this article.

## Supplementary information

Supplementary Information

Reporting Summary

Source Data

## Data Availability

Large data sets were not used in the manuscript. Raw data of different plots are provided in the Source Data file.
