# Supplementary material for: Rho-dependent transcription termination in bacteria recycles RNA polymerases stalled at DNA lesions
Source: Nat Commun. 2019 Mar 14;10:1207. doi: 10.1038/s41467-019-09146-5 (PMC6418286; doi:10.1038/s41467-019-09146-5)
Supplement: Supplementary file 1 — Supplementary Information [file 41467_2019_9146_MOESM1_ESM.pdf]

SUPPLEMENTARY INFORMATION

SUPPLEMENTARY FIGURES 1 to 6.

Rho-dependent transcription termination in bacteria recycles RNA polymerases stalled at the DNA lesions.

Jain et al.

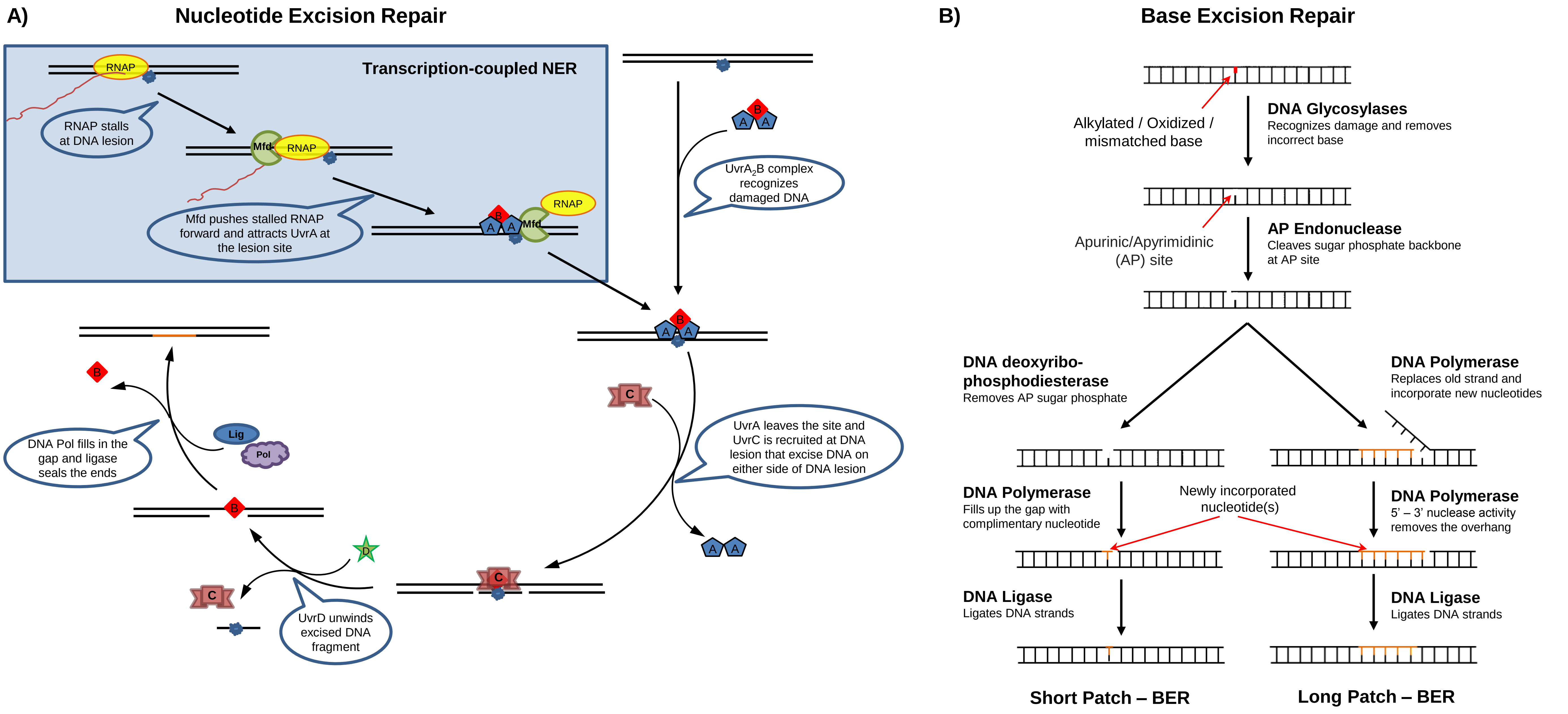

Supplementary Figure 1: Bacterial DNA Repair pathways: A) Steps involved in nucleotide excision repair pathway. Steps in the box represent the transcription-coupled steps (TCR). The stoichiometry of UvrA and UvrB during TCR and GGR is clearly known. B) Base excision repair pathway wherein small single base DNA lesions are repaired. The major BER pathway, Short patch BER, repairs DNA strand by replacing a single nucleotide whereas alternation long patch BER occurs by newly synthesizing relatively longer DNA strand.

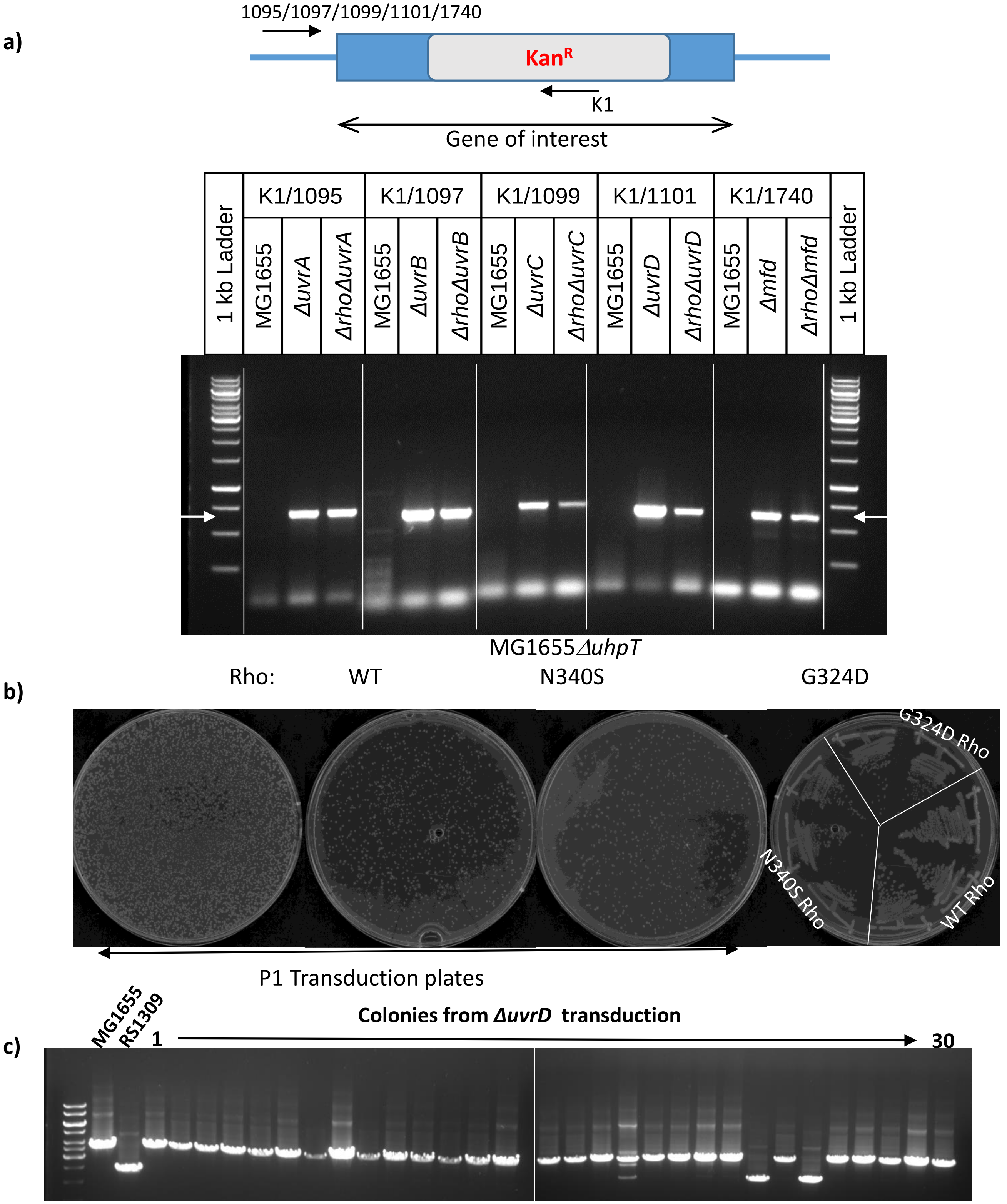

Supplementary Figure 2: a) *Specificity of the gene deletions***.** PCR was performed on the MG1655 strains having deletions in different NER genes as indicated. Primer pairs used are indicated. The location of the primer pairs are shown in the cartoon above the gel. NER genes (*uvrA, uvrB, uvrC, uvrD* and *Mfd*) were deleted by P1 transduction of RS1309 (*E. coli* MG1655 *Δrho Δrac*) with the P1 lysate prepared on the of the keio strains for respective genes. P1 transduction replaced the gene of interest with Kan^R cassette. Formation of the PCR products (arrow) is the indication that the Kanamycin-cassette has been inserted into the gene of interest only. MG1655 (Lane 2,5,8,11,14) as negative control does not amplify any fragment with these primer sets. **b**) *Effect of an unrelated gene***:** *uhpT,* an unrelated gene, is transduced into the MG1655 strains expressing either WT or mutant Rho proteins. Colonies from transduction plates were also streaked as shown in the right most panel. No significant synthetic defects were visible. **c**) *Checking of rho-uvrD co-transduction:* Colony PCR with primer pair RS1743 and RS1744 amplifies a region flanking the *rho*. MG1655 with WT *rho* generates a fragment of 3.6 kb (lane 2) whereas in the *rho* deletion strain (RS1309), this fragment size is smaller ~2.4 kb (Lane 3). Various uvrD tranductants (Colony no. 1 to 30) showed that only colony numbers 23 and 25 were *Δrho,* whereas in all the other colonies *rho* gets co-transformed with *uvrD::Kan^R*.

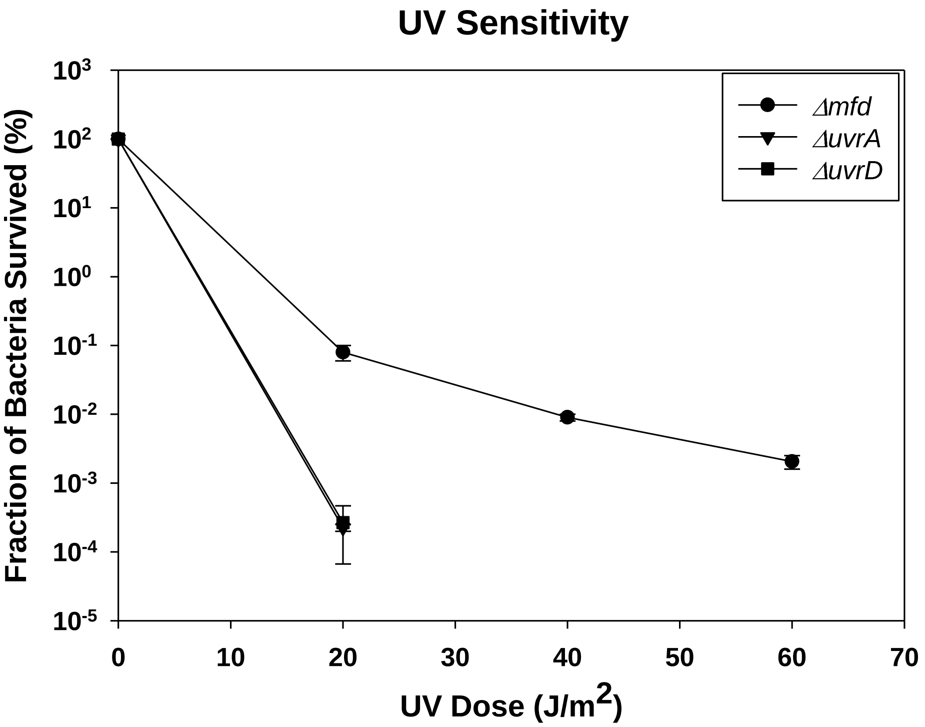

Supplementary figure 3: Comparison of UV sensitivity of the MG1655 strains deleted for either *uvrA* or *uvrD* or *mfd.* Note that the sensitivity plots of UvrA and UvrD are overlapped.

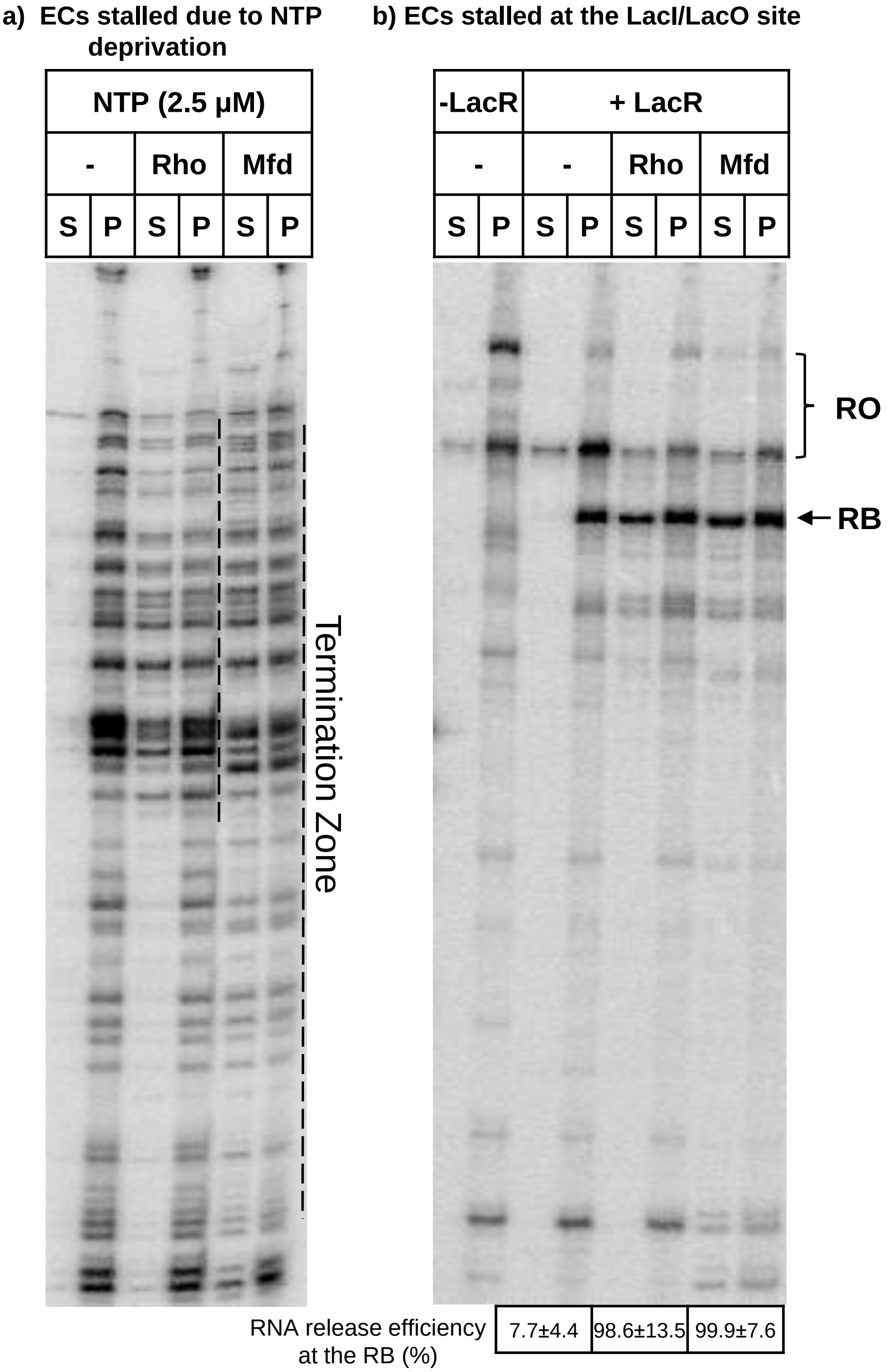

Supplemeentary figure 4: *Rho-mediated RNA release of RB complexes*. Autoradiogram showing the Rho and Mfd mediated RNA release from the ECs stalled due to NTP deprivation **(A)** and at the LacI/LacO site **(B)**. In **(A)** transcription reaction was chased with limited amount of nucleotides (2.5 µM). Zones of termination are indicated by the dotted lines. In **(B)** *in vitro* transcription performed on the immobilized DNA template carrying *lacO* site to bind the Lac repressor (LacI). LacI was added to the transcription reaction before formation of the $EC_{23.}$ Transcripts that reached the end of the template are shown as run-off (RO) while those blocked by LacI are indicated by RB (Road-blocked). Values at the bottom indicate RNA released (%) from the RB by Rho or Mfd.

a)

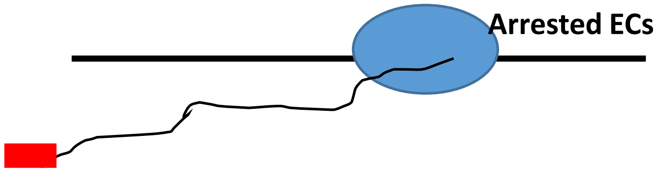

5 Cs are radiolabelled at the 5'-end of the nascent RNA of the arrested ECs. Hence, 5'RNA fragments could be visible after the GreB cleavage and not the 3'ones.

b) c)

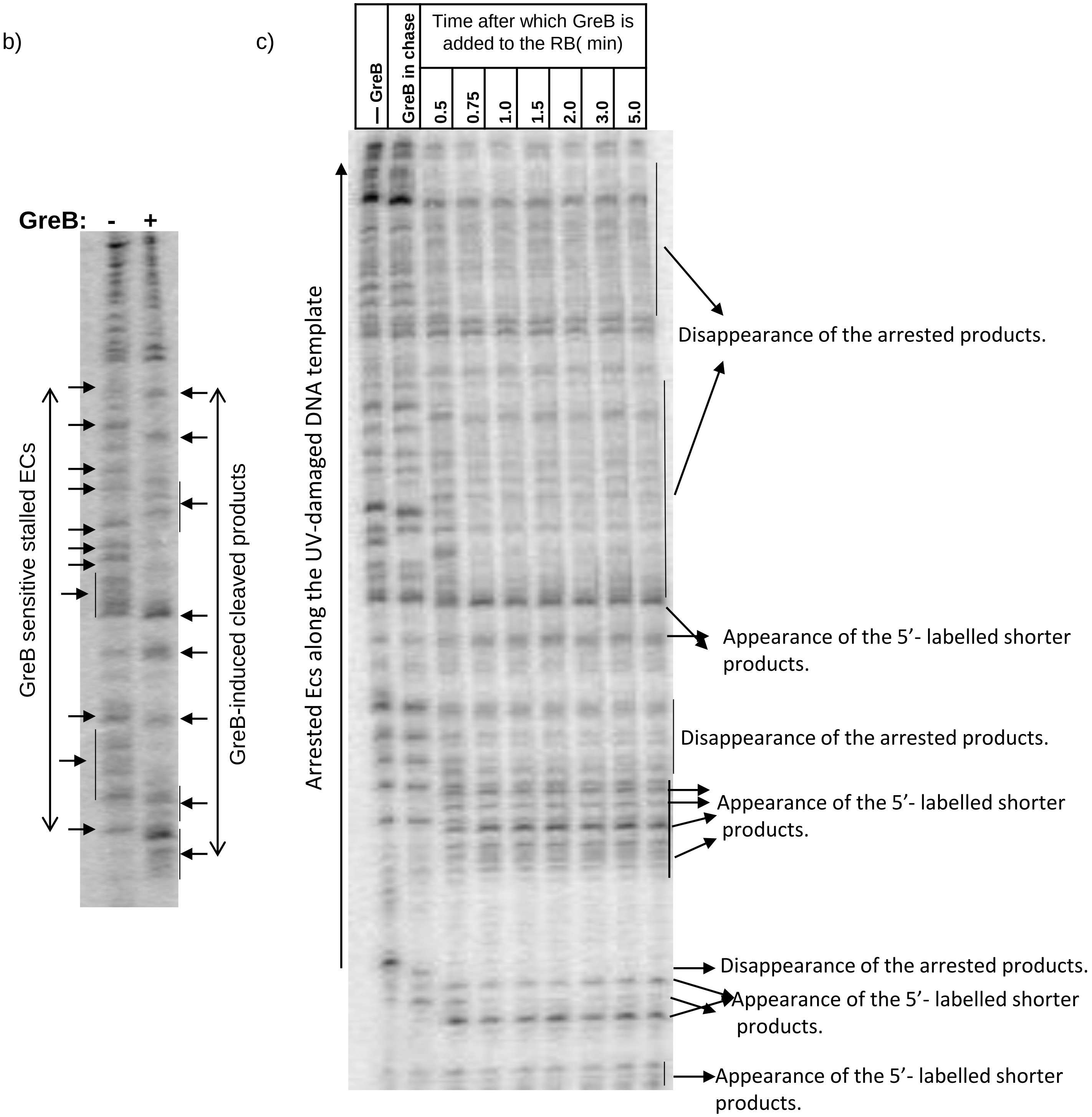

Supplementary figure 5: *Nature of stalled complexes at the DNA lesions*: **A)** Cartoon showing 5'-radiolabeled mRNA of the arrested ECs at the the T-T dimers along the template. **B)** Autoradiogram showing ECs stalled at the T-T dimers on the transcription template formed by UV exposure. Addition of GreB produces cleaved releases transcripts with reduced size (lane 2) as compared to those in the absence of GreB (Lane1) indicating that the ECs stalled at T-T dimers are backtracked**. C)** Appearances and disappearances of different RNA fragments from the arrested ECs are shown upon addition of the 500 nM GreB at the indicated time points after formation of the arrested ECs. Other reaction conditions are same as described in figures 5.

a) RNA release kinetics on UV-irradiated DNA (Template same as in figure 5):

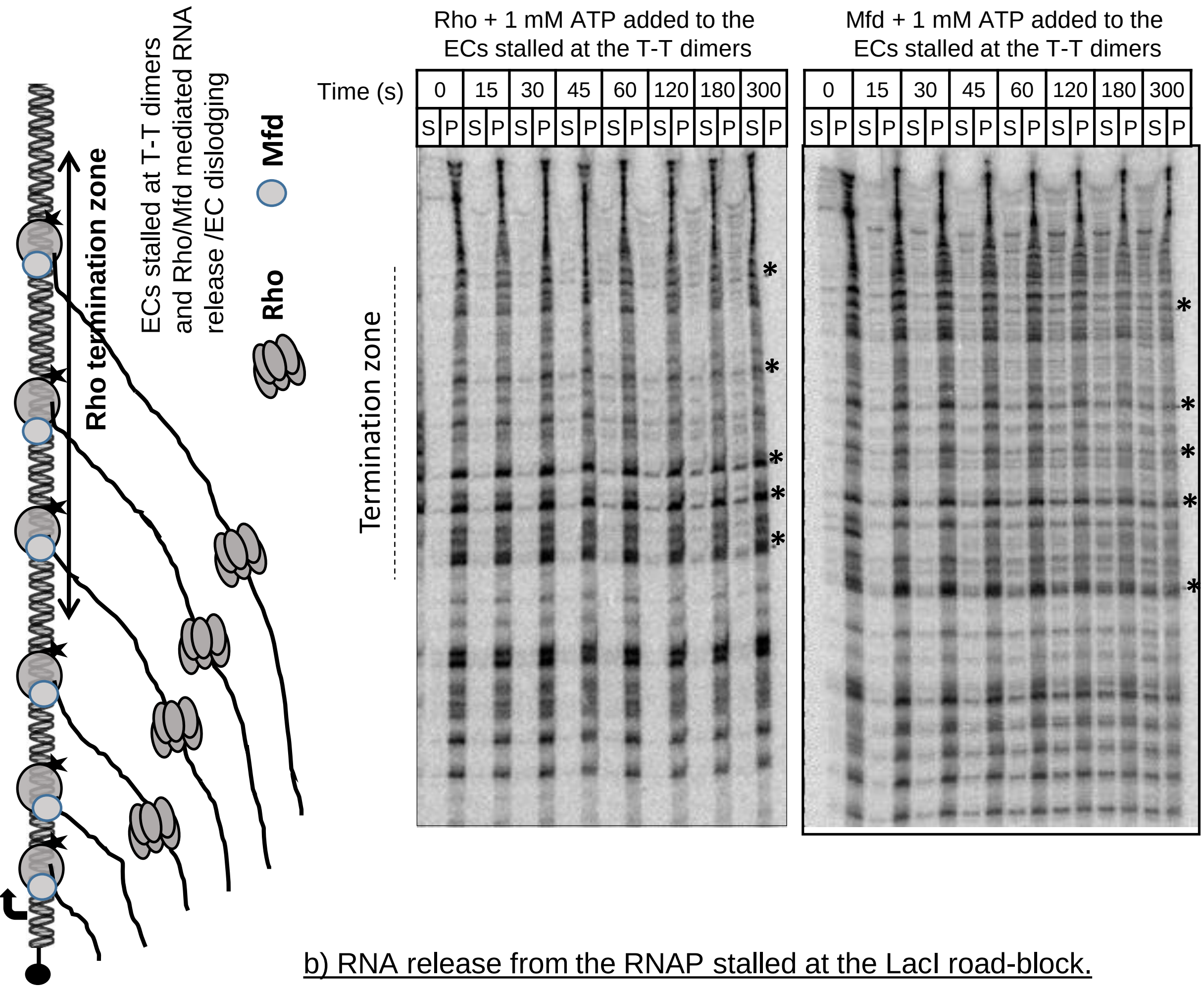

b) RNA release from the RNAP stalled at the LacI road-block.

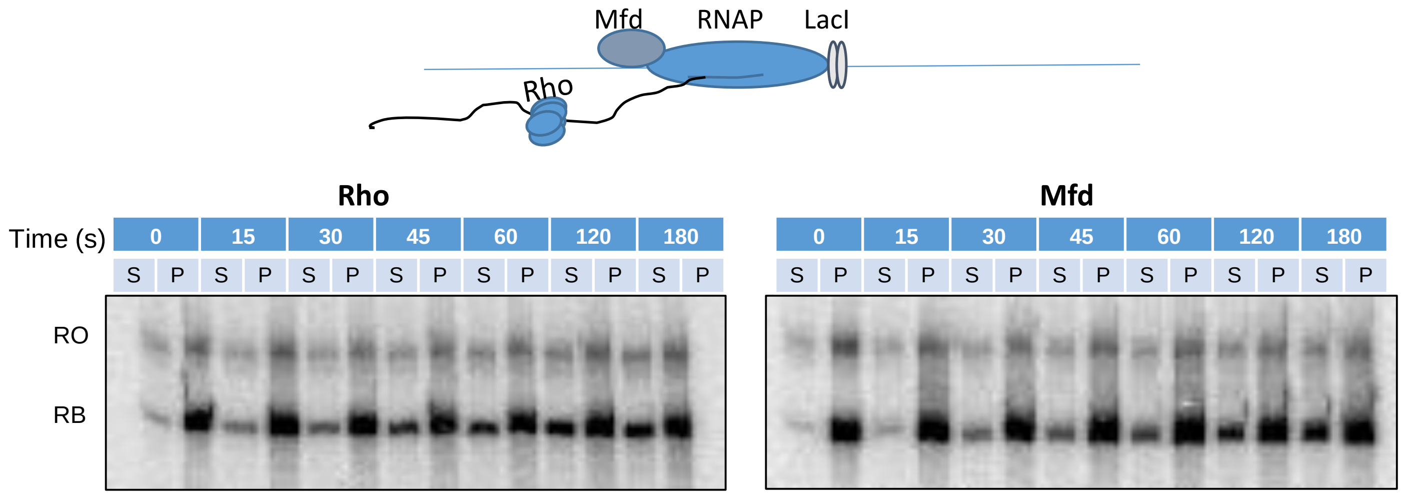

Supplementray figure 6: *RNA-release kinetics by Rho and Mfd.* a**)** Autoradiogram showing Rho- and Mfd-induced RNA release from the ECs stalled at T-T dimers. Efficiency of RNA released was calculated by the average of intensity of multiple bands (indicated by *) in the Rho termination zone. The kinetics curves are shown in figure 6A. Schematic representation of the stalled ECs are shown on the left of the autoradiogram. **b)** Cartoon shows action of Rho or Mfd on the ECs blocked by LacI. Autoradiogram showing the Rho- and Mfd-induced RNA release kinetics from the ECs stalled at LacI. Efficiency of RNA released was calculated by the intensity of the band indicated as RB from the samples collected at different time points. Rho showed higher efficiency than the Mfd as indicated by the curve in figure 6B.
